# Factors Associated with Participation, Active Refusals and Reasons for Not Taking Part in a Mortality Followback Survey Evaluating End-of-Life Care

**DOI:** 10.1371/journal.pone.0146134

**Published:** 2016-01-08

**Authors:** Natalia Calanzani, Irene J Higginson, Jonathan Koffman, Barbara Gomes

**Affiliations:** 1 King’s College London, Cicely Saunders Institute, Department of Palliative Care, Policy & Rehabilitation, London, United Kingdom; 2 University of Edinburgh, The Usher Institute of Population Health Sciences and Informatics, Centre for Population Health Sciences, Medical School, Edinburgh, United Kingdom; Rutgers University, UNITED STATES

## Abstract

**Background:**

Examination of factors independently associated with participation in mortality followback surveys is rare, even though these surveys are frequently used to evaluate end-of-life care. We aimed to identify factors associated with 1) participation versus non-participation and 2) provision of an active refusal versus a silent refusal; and systematically examine reasons for refusal in a population-based mortality followback survey.

**Methods:**

Postal survey about the end-of-life care received by 1516 people who died from cancer (aged ≥18), identified through death registrations in London, England (response rate 39.3%). The informant of death (a relative in 95.3% of cases) was contacted 4–10 months after the patient died. We used multivariate logistic regression to identify factors associated with participation/active refusals and content analysis to examine refusal reasons provided by 205 nonparticipants.

**Findings:**

The odds of partaking were higher for patients aged 90+ (AOR 3.48, 95%CI: 1.52–8.00, ref: 20–49yrs) and female informants (AOR 1.70, 95%CI: 1.33–2.16). Odds were lower for hospital deaths (AOR 0.62, 95%CI: 0.46–0.84, ref: home) and proxies other than spouses/partners (AORs 0.28 to 0.57). Proxies of patients born overseas were less likely to provide an active refusal (AOR 0.49; 95% CI: 0.32–0.77). Refusal reasons were often multidimensional, most commonly study-related (36.0%), proxy-related and grief-related (25.1% each). One limitation of this analysis is the large number of nonparticipants who did not provide reasons for refusal (715/920).

**Conclusions:**

Our survey better reached proxies of older patients while those dying in hospitals were underrepresented. Proxy characteristics played a role, with higher participation from women and spouses/partners. More information is needed about the care received by underrepresented groups. Study design improvements may guide future questionnaire development and help develop strategies to increase response rates.

## Introduction

Mortality followback surveys with bereaved relatives are used in several countries such as US [1], the UK [2], Japan [3] and Italy [4] to evaluate end-of-life care, but the method faces ethical and methodological challenges [5]. From an ethical perspective, sensitive planning is required to avoid distress to a population that can be vulnerable [6–8]. Researchers should make sure they maximise research benefits, while minimising/not causing harm to participants [8–10]. From a methodological perspective, a core concern is how to increase participation to avoid low response rates (RRs).

Low RRs can result in nonresponse bias when there are systematic differences between participants and nonparticipants and these are correlated with the variables of interest in a study [11–13]. Methods to increase the odds of response in surveys have been widely investigated [12,14–19]. However, nonparticipation remains an unavoidable reality. Furthermore, comparing participants to nonparticipants is the exception rather than the rule; information on nonparticipants is rare in mortality followback surveys [13,20]. When this analysis is conducted, the adopted statistical methods are usually not consistent [21–24] or not clear [1,25–27]. It is therefore not surprising that factors such as ethnicity [21,28], age [21,27,29], relationship to deceased [21], gender [29], place of death [22], interval from time to death [22] and social deprivation [29] do not show a consistent association with participation across studies with bereaved relatives, even when the studied population and survey methods used are similar. Crucially, the use of multivariate analysis to adjust for potential confounders has been rarely applied to examine nonresponse in this type of survey.

In addition to methodological issues, a key ethical concern in mortality followback surveys is whether participants and nonparticipants are being harmed by research. Current evidence suggests that most participants find it beneficial to take part and that many feel good about having the opportunity to help others in similar situations [30–34]. Those who do not participate, however, might have different views. A few studies have investigated the reasons why some bereaved relatives do not take part in mortality followback surveys. Their results suggest that grief and strong emotions [22,35–39] are common refusal reasons. Others include being “too busy” [36,37,39–42], not knowing the deceased [35,43], being too ill [36,39], or not being interested [39–42]. A better understanding of why people refuse to take part is still needed, as the knowledge can help identify areas in need of improvement in order to increase RRs.

This study aims to determine factors associated with participation in a cancer mortality followback postal survey and to systematically investigate reasons for refusing to take part. It also determines factors associated with providing an active refusal as opposed to a silent refusal (i.e. those who did not contact the research team to refuse participation and did not return a completed questionnaire). With this knowledge, we are able to identify underrepresented groups and ways to improve overall response in postal bereavement surveys.

## Materials and Methods

### 1 Study design and setting

The QUALYCARE study was a mortality followback postal survey aiming to examine variations in the quality and costs of end-of-life care, preferences and palliative outcomes associated with death at home in cancer. The study took place in four health regions in London, United Kingdom (UK) with contrasting cancer home death rates and deprivation levels. These were chosen based on ecological analysis. The study protocol can be found elsewhere [44]. This analysis of the QUALYCARE dataset focuses on non-response, a key component of the study which was pre-specified in the QUALYCARE protocol.

### 2 Participants and sampling

The study was conducted with proxies for people ≥18 who lived in one of the four health regions and died from cancer between 5^th^ March 2009 and 4^th^ March 2010 (one year period). The deceased were identified from death registrations and the proxy was the person who registered the death (i.e. the informant of death). The Office for National Statistics (ONS) conducted the sampling in two waves (Nov 2009 and May 2010). They selected all deaths registered 4–10 months prior to when the survey would be sent by post and screened for further eligibility criteria (deceased aged ≥18, resident in one of the four included health regions, cancer as the underlying cause of death). The sample was stratified by health region and place of death. For each health region we included all deaths that took place at home, in hospices and in nursing homes. We then drew a random sample of 150 hospital deaths per health region or took all hospital deaths if the number was below 150. The latter happened in the two smallest regions. A random sample of hospital deaths was drawn in the larger health regions to ensure that hospital deaths did not drown out deaths taking place in other settings (the latter were all included to ensure they were well represented within the sample size required). In 2009 and 2010 over 40% of cancer deaths (44.0% and 42.0% respectively) in England and Wales occurred in a public hospital [45,46]. Hospital cancer deaths are also more likely in urban areas such as London [47]. Cases where the death took place in other places or when the place of death was unknown, and deaths registered by coroners were excluded. Questionnaire packs were sent in two waves (January and July 2010) to cover the one year period.

The sample size needed for the original objectives of the QUALYCARE study was calculated based on Altman’s methods [48]; a total sample of ~500 participants was needed to enable powered comparisons on preferences for place of death, help of community nursing and satisfaction with GP care by place of death for the most detailed level of analysis (a case-control of home vs. hospital deaths, which required ~350 participants). The RR was estimated at 38% with an extra 10% of missing data. Further information is available elsewhere [44].

### 3 Recruitment

The ONS sent each of the eligible participants a questionnaire pack by post following an “opt-out” recruitment approach. This was done on behalf of the research team as it did not have access to patients or informants of death’s names and addresses. The pack included a personalised invitation letter (with the potential participant’s name and address); an information leaflet tailored to the specific health region explaining the purpose of the study and providing the research team’s address and telephone number; a reply slip and a small freepost envelope (addressed to the ONS) for people to refuse to take part if that was their wish; a leaflet produced by the Royal College of Psychiatrists with information about bereavement and sources of support [49]; the study questionnaire (numbered, gender-specific and with a coloured cover) and a large freepost envelope for returning the questionnaire to the research team.

Receiving a completed questionnaire was considered as consent to participate. Up to two reminders were sent to people who did not respond at two and four weeks after the initial posting; the second reminder included another copy of the questionnaire. Those who had sent their refusals were coded as nonparticipants and did not receive any further reminders.

### 4 Data collection

Data were collected by using a purpose-built 44-page questionnaire (available from the research team), a reply slip in the questionnaire pack and a standardised call recording form which was completed for each telephone call answered by the research team (S1 and S2 Figs). Further pseudo-anonymised socio-demographic data were provided by the ONS.

#### 4.1 The questionnaire

QUALYCARE followed methods by Cartwright, McCarthy and Addington-Hall for national surveys in England on experiences of care in the last year of life [44]. The questionnaire included four other tools: the Client Service Receipt Inventory (CSRI), the Palliative care Outcome Scale (POS), a health status measure (EuroQoL EQ-5D-3L) and the Texas Revised Inventory of Grief (TRIG). Relevant socio-demographic information about the patient and the proxy was collected in the questionnaire. The questionnaire was piloted using cognitive interviewing with 20 bereaved relatives recruited via the palliative care department of a hospital in London [34,50,51]. This helped to refine the methods and improve the questionnaire.

#### 4.2 Reply slip and calls

The reply slip had a box for potential participants to tick in case they did not wish to take part. It also stated that, although not required, it would be helpful for the researchers to know why they decided not to participate. The slip had space for open comments and no reasons were suggested. The research team was also available over the telephone during working hours to answer any queries.

#### 4.3 Death registration information

The ONS provided the research team with a pseudo-anonymised and encrypted dataset for the entire sample. This dataset had information on patient’s region of residence, patient’s age, days and months from both actual death and registration of death until the date when the questionnaire was sent, place of death, patient’s gender, patient’s country of birth, proxy’s relationship to the deceased, and underlying cause of death (based on the 10^th^ revision of the International Classification of Diseases—ICD-10). Underlying cause of death referred to cancer deaths only (C00 to D48)) [52].

The ONS also provided overall Index of Multiple Deprivation (IMD) scores (2010) characterising the patient’s area of residence (according to Lower layer Super Output Areas). The overall IMD score is a single measure of multiple deprivation; it is a weighted area level aggregation of specific dimensions of deprivation (income, employment, education, health, among others) [53]. These were provided as overall scores and deciles, which were then recoded into quintiles. The first quintile represents the most deprived areas and the fifth quintile represents the least deprived areas.

### 5 Ethics

The study was approved by a NHS Research Ethics Committee (REC), namely the London—Dulwich REC, formerly King’s College London REC (ref no.:09/H0808/85). The R&D offices of the health regions included in the study were notified about the study. No approval was needed as the participants were not recruited through National Health Services. Access to and handling of the anonymised death registration data provided by the ONS was governed by a Data Access Agreement signed by the ONS and the Cicely Saunders Institute (where the researchers were based).

Returning a completed questionnaire was considered as consent to participate. The London-Dulwich REC approved this consent procedure. The research team had no access to participant identifiable data (this was only accessed by the ONS) unless this was willingly provided by participants at the end of the questionnaire (where they were asked if they wished to receive a short summary of the study results and if they were happy to be contacted by the research team).

The ‘opt out’ approach to recruitment was decided following debates with experts in ethics, end of life care bereavement researchers and clinicians, consultation of national guidance, analysis of previous studies and of findings from the pilot study. Participants were informed in the invitation letter that the study had an ‘opt-out’ approach, and there were detailed procedures to ensure confidentiality and security of personal data. Participants were assured in the invitation letter and also in the information leaflet that they were under no obligation to take part or answer any questions that they considered distressing. Procedures were followed to identify and handle distressed participants [44].

### 6 Statistical analysis

Descriptive statistics, univariate and multivariate analyses were performed with the software PASW Statistics for Windows 18.0 (IBM).

We report descriptive data on the number of completed questionnaires, cumulative RRs, distribution of RRs according to time of contact, reasons for refusing to take part in the study and socio-demographic characteristics from participants and nonparticipants. Response rates correspond to the number of returned questionnaires divided by the number of eligible people to whom questionnaires were sent by post.

Participants and nonparticipants were compared on socio-demographic variables by using descriptive statistics. We carried out univariate and multivariate logistic regression to identify factors associated with participation and factors associated with active refusal. Variables were chosen a priory based on factors which were previously associated with non-participation in the literature [21,22,27–29].

Our analyses included the variables health region and place of death to control for any design effect associated with the fact that our sample was stratified by these two variables. We calculated unadjusted and adjusted odds ratios (OR and AOR respectively) with 95% confidence intervals (CIs). We also evaluated how the multivariate models fit the observed data (ROC curve, Nagelkerke R^2^, Hosmer-Lemeshow goodness-of-fit test). All potential explanatory variables that we measured were included in the models and entered simultaneously. Tests were two-tailed and p<0.05 was deemed significant in the final regression models. Cases with missing data were excluded.

All reasons for refusal were independently read by two researchers (both psychologists) and analysed line by line using content analysis. An inductive coding frame with categories and subcategories was derived from the data. After agreement was reached for all categories and subcategories, the same researchers independently coded each refusal. Kappa statistics were used for each subcategory to verify the raters’ level of agreement. Strength of agreement was assessed following Landis and Koch guidelines (almost perfect agreement 0.81–1.00; substantial 0.61–0.80; moderate 0.41–0.60; poor <0.00) [54]. All discrepancies were discussed and a final agreement was obtained by consensus in these cases.

## Results

### 1 Response rates

**Fig 1:**
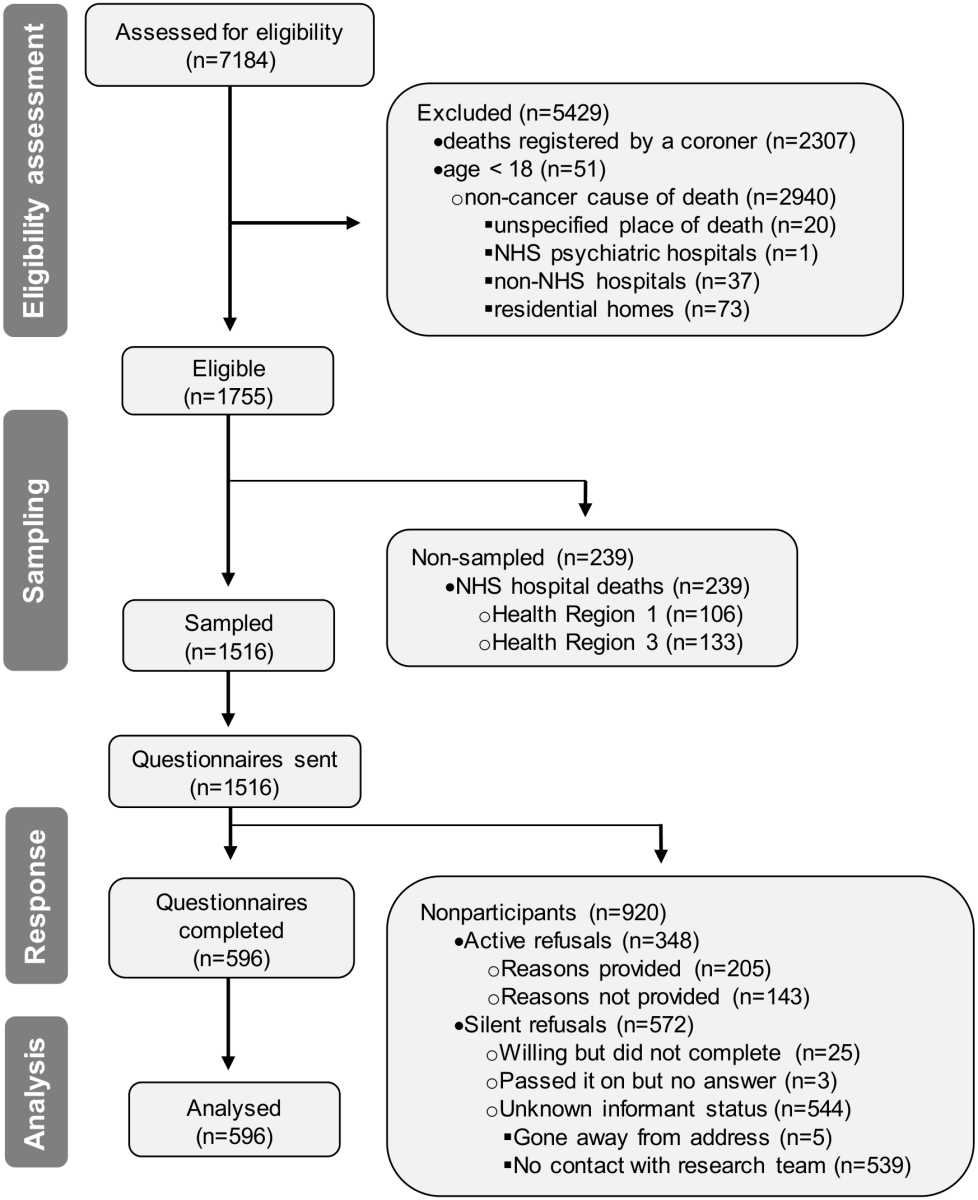
QUALYCARE flow diagram. NHS, National Health Services.

**Fig 2:**
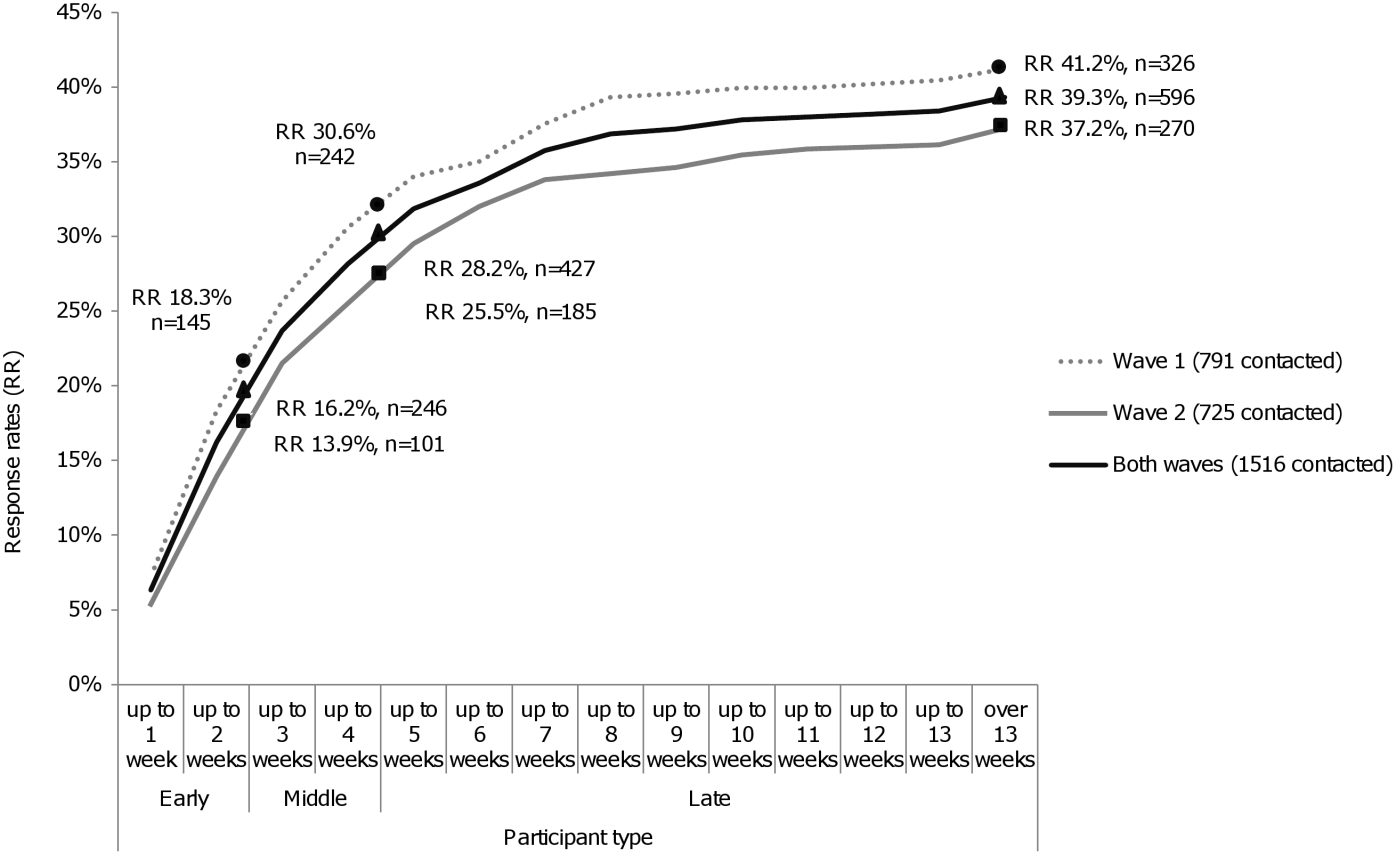
Cumulative response rates by wave (1, 2 and both) and type of participant (early, middle and late).

We sent 1516 questionnaire packs and received 596 completed questionnaires, resulting in a 39.3% RR (Fig 1). We received 71.6% (n = 427) of the completed questionnaires during the first four weeks after these were sent by post (Fig 2). All but six questionnaires were returned 4 to 8 months (n = 324; 54.4%), 9 to 10 months (n = 169; 28.4%) or 11 to 12 months (n = 97; 16.3%) after the patient died. The median number of days from death to the return of a completed questionnaire was 232.50 days (interquartile range: 191.25–281.00). Response rates varied from 32.4% in heath region 2 to 46.6% in health region 1, and were highest in Wave 1 (41.2%) compared to Wave 2 (37.2%).

### 2 Sample characteristics

60.5% of participants were women and the majority were either the patient’s sons or daughters (46.2%) or their spouse/partner (37.6%). There was a similar proportion of male (51.5%) and female (48.5%) patients represented in the survey. Patient’s median age at death was 77.0 [interquartile range 66.0–84.0]. The most common types of cancer were digestive (27.9%) and respiratory or intra-thoracic (21.5%) (Table 1).

**Table 1:** Characteristics of participants and nonparticipants.

Characteristics*	All sampled deaths (n = 1516)	A) Participants (n = 596)	B) Nonparticipants (n = 920)
Patient gender			
Male	775 (51.1%)	307 (51.5%)	468 (50.9%)
Female	741 (48.9%)	289 (48.5%)	452 (49.1%)
Patient age			
Median age (IQR)	76.0 (66.0–83.0)	77.0 (66.0–84.0)	75.5 (66.0–82.0)
20–49	72 (4.7%)	23 (3.9%)	49 (5.3%)
50–59	142 (9.4%)	50 (8.4%)	92 (10.0%)
60–69	264 (17.4%)	118 (19.8%)	146 (15.9%)
70–79	455 (30.0%)	151 (25.3%)	304 (33.0%)
80–89	490 (32.3%)	205 (34.4%)	285 (31.0%)
90+	93 (6.1%)	49 (8.2%)	44 (4.8%)
Patient’s country of birth			
Born in the UK or Ireland	1223 (81.0%)	497 (83.4%)	726 (79.4%)
Born overseas^†^	287 (19.0%)	99 (16.6%)	188 (20.6%)
IMD score for lower super output area where patient lived			
Median IMD score (IQR)	17.3 (9.5–30.4)	14.4 (7.8–27.0)	19.1 (10.1–32.2)
5^th^ Quintile (least deprived)	329 (21.7%)	161 (27.0%)	168 (18.3%)
4^th^ Quintile	275 (18.1%)	120 (20.1%)	155 (16.8%)
3^rd^ Quintile	260 (17.2%)	94 (15.8%)	166 (18.0%)
2^nd^ Quintile	363 (23.9%)	136 (22.8%)	227 (24.7%)
1^st^ Quintile (most deprived)	289 (19.1%)	85 (14.3%)	204 (22.2%)
Health region where patient lived			
Health region 1	487 (32.1%)	227 (38.1%)	260 (28.3%)
Health region 2	293 (19.3%)	95 (15.9%)	198 (21.5%)
Health region 3	481 (31.7%)	184 (30.9%)	297 (32.3%)
Health region 4	255 (16.8%)	90 (15.1%)	165 (17.9%)
Type of cancer (underlying cause of death)			
Digestive organs	422 (27.8%)	166 (27.9%)	256 (27.8%)
Respiratory and intra-thoracic organs	327 (21.6%)	128 (21.5%)	199 (21.6%)
Eye, brain and other parts of CNS	46 (3.0%)	27 (4.5%)	19 (2.1%)
Breast	129 (8.5%)	51 (8.6%)	78 (8.5%)
Lymphoid/haematopoietic/related tissue	111 (7.3%)	38 (6.4%)	73 (7.9%)
Male genital organs	117 (7.7%)	42 (7.0%)	75 (8.2%)
Female genital organs	65 (4.3%)	26 (4.4%)	39 (4.2%)
Urinary tract	84 (5.5%)	39 (6.5%)	45 (4.9%)
Lip, oral cavity and pharynx	23 (1.5%)	6 (1.0%)	17 (1.8%)
Melanoma and skin	26 (1.7%)	12 (2.0%)	14 (1.5%)
Uncertain/unspecified/other	166 (11.1%)	61 (10.2%)	105 (11.5%)
Place of death			
Home	368 (24.3%)	175 (29.4%)	193 (21.0%)
Hospital	513 (33.8%)	177 (29.7%)	336 (36.5%)
Hospice	512 (33.8%)	199 (33.4%)	313 (34.0%)
Nursing home	123 (8.1%)	45 (7.6%)	78 (8.5%)
Gender of proxy			
Male	649 (47.4%)	220 (39.5%)	429 (52.8%)
Female	720 (52.6%)	337 (60.5%)	383 (47.2%)
Proxy’s relationship to patient			
Spouse or partner	413 (29.9%)	211 (37.6%)	202 (24.7%)
Son/daughter	688 (49.9%)	259 (46.2%)	429 (52.4%)
Brother/sister	108 (7.8%)	37 (6.6%)	71 (8.7%)
Mother/father	17 (1.2%)	8 (1.4%)	9 (1.1%)
Niece/nephew	70 (5.1%)	23 (4.1%)	47 (5.7%)
Grandson/granddaughter	20 (1.4%)	6 (1.1%)	14 (1.7%)
Other	64 (4.6%)	17 (3.0%)	47 (5.7%)
Days death registration to contact			
Median days (IQR)	213.00 (167.25–258.00)	210.00 (168.00–256.00)	215.00 (166.00–258.00)
Days death to contact			
Median days (IQR)	213.00 (167.00–259.00)	209.00 (168.00–256.75)	215.00 (167.00–261.00)

*Abbreviations*: IQR, Interquartile range; IMD, Index of Multiple Deprivation; CNS, Central Nervous System.

*There were no missing data on patient’s gender, age, place of death, cancer type, region and IMD score. Missing data was 0.4% (country of birth), 9.0% (proxy’s relationship to patient), 9.7% (proxy’s gender) and 0.1% (days death to contact)

^†^ There were 76 overseas countries of birth, none with more than 19 counts.

### 3 Factors associated with participation

When comparing participants with nonparticipants using univariate statistics, there was an under-representation of people living in more deprived areas except for the 4^th^ quintile (OR ranged from 0.44 for the 1^st^ quintile to 0.63 for the 2^nd^ quintile) and deaths outside home (OR ranged from 0.58 for hospital to 0.70 for hospice) and cases in which the person who registered the death (proxy) was the patient’s son or daughter, brother or sister, niece/nephew or other (OR ranged from 0.35 for other to 0.58 for a son/daughter). On the other hand, we observed an over-representation of patients aged 90+ (OR 2.37; 95% CI:1.25–4.51) and proxies who were women (OR 1.72; 95% CI:1.38–2.14). There were no significant differences between participants and nonparticipants in patient’s country of birth, patient’s gender, type of cancer and days from death to contact by researchers (Table 2).

**Table 2:** Factors associated with participation.

Factors	Response rate	Univariate analysis		p-values	Multivariate analysis*		p-values
		n	OR (95% CI)		n	AOR (95% CI)	
Gender of patient							
Male	39.6%	775	*Ref*.	p = 0.807	697	*Ref*.	p = 0.628
Female	39.0%	741	0.98 (0.79–1.20)		672	1.07 (0.82–1.40)	
Age of patient							
20–49	31.9%	72	*Ref*.	p = 0.001	64	*Ref*.	p<0.001
50–59	35.2%	142	1.16 (0.63–2.12)		126	1.18 (0.56–2.49)	
60–69	44.7%	264	1.72 (0.99–2.99)		239	1.80 (0.88–3.65)	
70–79	33.2%	455	1.06 (0.62–1.80)		403	1.15 (0.57–2.32)	
80–89	41.8%	490	1.53 (0.91–2.60)		454	1.88 (0.93–3.81)	
90+	52.7%	93	2.37 (1.25–4.51)		83	3.48 (1.52–8.00)	
Patient’s country of birth							
Born in the UK or Ireland	40.6%	1223	*Ref*.	p = 0.056	1111	*Ref*.	p = 0.554
Born overseas	34.5%	287	0.77 (0.59–1.01)		258	0.91 (0.67–1.24)	
IMD 2010 score							
5^th^ Quintile (least deprived)	48.9%	329	*Ref*.	p<0.001	311	*Ref*.	p = 0.050
4^th^ Quintile	43.6%	275	0.81 (0.59–1.11)		259	0.79 (0.56–1.13)	
3^rd^ Quintile	36.2%	260	0.59 (0.42–0.82)		225	0.66 (0.45–0.97)	
2^nd^ Quintile	37.5%	363	0.63 (0.46–0.85)		329	0.73 (0.51–1.05)	
1^st^ Quintile (most deprived)	29.4%	289	0.44 (0.31–0.61)		245	0.50 (0.31–0.80)	
Health region where patient lived							
Health region 1	46.6%	487	*Ref*.	p<0.001	467	*Ref*.	p = 0.120
Health region 2	32.4%	293	0.55 (0.41–0.74)		249	0.84 (0.55–1.28)	
Health region 3	38.3%	481	0.71 (0.55–0.92)		444	0.76 (0.57–1.01)	
Health region 4	35.3%	255	0.63 (0.46–0.85)		209	1.09 (0.74–1.61)	
Type of cancer (underlying cause of death)							
Digestive organs	39.3%	422	*Ref*.	p = 0.222	379	*Ref*.	p = 0.421
Respiratory and intra-thoracic organs	39.1%	327	0.99 (0.74–1.33)		293	1.02 (0.73–1.42)	
Eye, brain and other parts of CNS	58.7%	46	2.19 (1.18–4.07)		43	1.97 (1.00–3.88)	
Breast	39.5%	129	1.01 (0.67–1.51)		120	0.98 (0.61–1.56)	
Lymphoid/haematopoietic/related tissue	34.2%	111	0.80 (0.52–1.24)		100	0.92 (0.56–1.49)	
Male genital organs	35.9%	117	0.86 (0.56–1.32)		105	0.75 (0.46–1.22)	
Female genital organs	40.0%	65	1.03 (0.60–1.75)		59	1.14 (0.62–2.08)	
Urinary tract	46.4%	84	1.34 (0.83–2.14)		76	1.35 (0.80–2.28)	
Lip, oral cavity and pharynx	26.1%	23	0.54 (0.21–1.41)		21	0.54 (0.20–1.50)	
Melanoma and skin	46.2%	26	1.32 (0.60–2.93)		25	1.35 (0.57–3.19)	
Uncertain/unspecified/other	36.7%	166	0.90 (0.62–1.30)		148	0.89 (0.59–1.35)	
Place of death							
Home	47.6%	368	*Ref*.	p = 0.001	355	*Ref*.	p = 0.020
Hospital	34.5%	513	0.58 (0.44–0.76)		456	0.62 (0.46–0.84)	
Hospice	38.9%	512	0.70 (0.54–0.92)		455	0.74 (0.55–1.00)	
Nursing home	36.6%	123	0.64 (0.42–0.97)		103	0.68 (0.42–1.10)	
Gender of proxy							
Male	33.9%	649	*Ref*.	p<0.001	649	*Ref*.	p<0.001
Female	46.8%	720	1.72 (1.38–2.14)		720	1.70 (1.33–2.16)	
Proxy’s relationship to patient							
Spouse or partner	51.1%	413	*Ref*.	p<0.001	413	*Ref*.	p<0.001
Son or daughter	37.6%	688	0.58 (0.45–0.74)		688	0.57 (0.43–0.75)	
Brother or sister	34.3%	108	0.50 (0.32–0.78)		108	0.63 (0.39–1.01)	
Parent	47.1%	17	0.85 (0.32–2.25)		17	1.14 (0.35–3.75)	
Niece/nephew	32.9%	70	0.47 (0.27–0.80)		70	0.38 (0.21–0.68)	
Grandchild	30.0%	20	0.41 (0.16–1.09)		20	0.29 (0.10–0.84)	
Other	26.6%	64	0.35 (0.19–0.62)		53	0.28 (0.14–0.56)	
Days from death to contact							
110–150	40.4%	240	*Ref*.	p = 0.251	215	*Ref*.	p = 0.755
151–180	36.3%	256	0.84 (0.59–1.21)		227	0.89 (0.60–1.34)	
181–210	45.6%	250	1.24 (0.86–1.77)		232	1.24 (0.83–1.85)	
211–240	38.3%	248	0.92 (0.64–1.32)		224	0.97 (0.65–1.46)	
241–270	39.2%	217	0.95 (0.65–1.38)		206	0.98 (0.65–1.49)	
271–300	34.5%	220	0.78 (0.53–1.14)		190	0.95 (0.62–1.46)	
301–330	42.9%	84	1.11 (0.67–1.83)		75	1.16 (0.65–2.05)	

*Abbreviations*: UK, United Kingdom; IMD, Index of Multiple Deprivation; CNS, Central Nervous System; OR, odds ratio; AOR, adjusted odds ratio; CI, confidence intervals

*All characteristics of the patients and the informants of death, including timing of contact were entered simultaneously in the regression model (N = 1369, excluding 147 cases with missing data, i.e. 9.7% of all 1516 cases).Model-fitting statistics: Hosmer and Lemeshow (*Χ*^*2*^ *= 7*.*674*, *p = 0*.*466*), Nagelkerke R2 (12.1%). 64.7% of the overall cases were correctly predicted (39.7% of participants and 81.9% of nonparticipants). AUC: 0.679.

Multivariate analysis using logistic regression showed that patient’s age, place of death, proxy’s gender and relationship to patient were independently associated with participation (Table 2). The odds of taking part were highest if the patient was aged 90+ (AOR 3.48; 95%CI:1.52–8.00) and the proxy was a woman (AOR1.70; 95%CI:1.33–2.16). The odds of being a participant were lowest if the patient died in hospital (AOR 0.62; 95%CI:0.46–0.84) and the proxy was not a spouse/partner (except for brother/sister and parents; AOR ranged from 0.28 for other to 0.57 for a son/daughter).

### 4 Active refusal and associated factors

From the 920 nonparticipants, 348 (37.8%) actively refused to take part (either by calling the team, sending back the entire pack, posting a letter or the reply slip). The remaining nonparticipants (n = 572) did not contact the research team to refuse participation and did not return a completed questionnaire (i.e. the silent refusals) (Table 3). These corresponded to 539 (94.2%) non-participants who did not contact the research team in any way, 25 (4.4%) who called the research team stating they would try to complete the questionnaire but never managed to do so, three (0.5%) who informed the research team that they had passed the questionnaire on to someone else but the research team never received them and five (0.9%) questionnaire packs which were returned unopened because addressee had moved away (Fig 1). Eight potential participants who had sent a reply slip refusing participation changed their minds and later sent a completed questionnaire by post (these were included only in the participants’ group).

**Table 3:** Characteristics of nonparticipants.

Characteristics*	All nonparticipants (n = 920)	Active refusals (n = 348)	Silent refusals (n = 572)
Gender of patient			
Male	468 (50.9%)	188 (54.0%)	280 (49.0%)
Female	452 (49.1%)	160 (46.0%)	292 (51.0%)
Patient age			
Median age (IQR)	75.5 (66.0–82.0)	77.0 (70.0–84.0)	75.0 (64.0.0–81.8)
20–49	49 (5.3%)	13 (3.7%)	36 (6.3%)
50–59	92 (10.0%)	26 (7.5%)	66 (11.5%)
60–69	146 (15.9%)	42 (12.1%)	104 (18.2%)
70–79	304 (33.0%)	121 (34.8%)	183 (32.0%)
80–89	285 (31.0%)	124 (35.6%)	161 (28.1%)
90+	44 (4.8%)	22 (6.3%)	22 (3.8%)
Patient’s country of birth			
Born in the UK or Ireland	726 (79.4%)	298 (85.9%)	428 (75.5%)
Born overseas^†^	188 (20.6%)	49 (14.1%)	139 (24.5%)
IMD score for lower super output area where patient lived			
Median IMD score (IQR)	19.1 (10.1–32.2)	16.3 (9.2–30.4)	21.6 (10.9–33.7)
5^th^ Quintile (least deprived)	168 (18.3%)	78 (22.4%)	90 (15.7%)
4^th^ Quintile	155 (16.8%)	62 (17.8%)	93 (16.3%)
3^rd^ Quintile	166 (18.0%)	68 (19.5%)	98 (17.1%)
2^nd^ Quintile	227 (24.7%)	75 (21.6%)	152 (26.6%)
1^st^ Quintile (most deprived)	204 (22.2%)	65 (18.7%)	139 (24.3%)
Health region where patient lived			
Health region 1	260 (28.3%)	114 (32.8%)	146 (25.5%)
Health region 2	198 (21.5%)	64 (18.4%)	134 (23.4%)
Health region 3	297 (32.3%)	116 (33.3%)	181 (31.6%)
Health region 4	165 (17.9%)	54 (15.5%)	111 (19.4%)
Type of cancer (underlying cause of death)			
Digestive organs	256 (27.8%)	98 (28.2%)	158 (27.6%)
Respiratory and intra-thoracic organs	199 (21.6%)	74 (21.3%)	125 (21.9%)
Eye, brain and other parts of CNS	19 (2.1%)	7 (2.0%)	12 (2.1%)
Breast	78 (8.5%)	28 (8.0%)	50 (8.7%)
Lymphoid/haematopoietic/related tissue	73 (7.9%)	22 (6.3%)	51 (8.9%)
Male genital organs	75 (8.2%)	30 (8.6%)	45 (7.9%)
Female genital organs	39 (4.2%)	11 (3.2%)	28 (4.9%)
Urinary tract	45 (4.9%)	19 (5.5%)	26 (4.5%)
Lip, oral cavity and pharynx	17 (1.8%)	3 (0.9%)	14 (2.4%)
Melanoma and skin	14 (1.5%)	6 (1.7%)	8 (1.4%)
Uncertain/unspecified/other	105 (11.5%)	50 (14.4%)	55 (9.6%)
Place of death			
Home	193 (21.0%)	63 (18.1%)	130 (22.7%)
Hospital	336 (36.5%)	135 (38.8%)	201 (35.1%)
Hospice	313 (34.0%)	118 (33.9%)	195 (34.1%)
Nursing home	78 (8.5%)	32 (9.2%)	46 (8.0%)
Gender of proxy			
Male	429 (52.8%)	129 (43.0%)	300 (58.6%)
Female	383 (47.2%)	171 (57.0%)	212 (41.4%)
Proxy’s relationship to patient			
Spouse or partner	202 (24.7%)	110 (36.3%)	92 (17.8%)
Son/daughter	429 (52.4%)	116 (38.3%)	313 (60.7%)
Brother/sister	71 (8.7%)	31 (10.2%)	40 (7.8%)
Mother/father	9 (1.1%)	3 (1.0%)	6 (1.2%)
Niece/nephew	47 (5.7%)	24 (7.9%)	23 (4.5%)
Grandson/granddaughter	14 (1.7%)	4 (1.3%)	10 (1.9%)
Other	47 (5.7%)	15 (5.0%)	32 (6.2%)
Days death registration to contact			
Median days (IQR)	215.00 (166.00–258.00)	215.50 (165.25–258.00)	213.00 (167.00–259.50)
Days death to contact			
Median days (IQR)	215.00 (167.00–261.00)	216.00 (166.25–260.00)	213.00 (167.00–262.00)

*Abbreviations*: IQR, Interquartile range; IMD, Index of Multiple Deprivation; CNS, Central Nervous System

*There were no missing data on patient’s gender, age, place of death, cancer type, region and IMD score. Missing data was 0.7% (country of birth), 11.0% (proxy’s relationship to patient), 11.7% (proxy’s gender) and 0.1% (days death to contact)

^†^ There were 60 overseas countries of birth, none with more than 16 counts.

Similarly to the comparison between participants and nonparticipants, when comparing those who provided active refusals with the silent refusals using univariate analysis, proxies of patients who lived in the two most deprived areas (OR 0.55 for the 1^st^ quintile and 0.57 for the 2^nd^ quintile) or who lived in health region 2 (OR 0.62; 95% CI:0.42–0.91) or health region 4 (OR 0.62; 95% CI:0.41–0.93), proxies who were the patient’s son/daughter (OR 0.31; 95% CI:0.22–0.44) or other (OR 0.40; 95% CI:0.20–0.78) provided active refusals less often. Informants of patients aged 90+ (OR 2.77; 95% CI:1.16–6.59) and 80–89 (OR 2.10; 95% CI:1.07–4.13) more often actively refused to take part compared to informants of patients aged 20–49; female proxies were also more likely to provide active refusals (OR 1.84; 95% CI:1.38–2.45). Proxies of patients who were born overseas provided active refusals less often than proxies of patients who were born in the country (OR 0.51; 95% CI:0.35–0.72) (Table 4).

**Table 4:** Factors associated with giving an active refusal.

Factors	Active refusal rate	Univariate analysis		p-values	Multivariate analysis*		p-values
		n	OR (95% CI)		n	AOR (95% CI)	
Gender of patient							
Male	40.0%	468	*Ref*.	p = 0.135	407	*Ref*.	p = 0.940
Female	35.2%	452	0.82 (0.62–1.07)		405	1.15 (0.70–1.47)	
Age of patient							
20–49	26.5%	49	*Ref*.	p = 0.003	41	*Ref*.	p<0.001
50–59	28.3%	92	1.09 (0.50–2.38)		81	1.30 (0.48–3.47)	
60–69	28.8%	146	1.12 (0.54–2.32)		132	1.97 (0.77–5.02)	
70–79	39.5%	304	1.81 (0.92–3.55)		262	3.42 (1.39–8.42)	
80–89	43.2%	285	2.10 (1.07–4.13)		258	4.43 (1.76–11.13)	
90+	50.0%	44	2.77 (1.16–6.59)		38	6.59 (2.12–20.56)	
Patient’s country of birth							
Born in the UK or Ireland	41.0%	726	*Ref*.	p = 0.001	646	*Ref*.	p = 0.002
Born overseas	26.1%	188	0.51 (0.35–0.72)		166	0.49 (0.32–0.77)	
IMD 2010 score							
5^th^ Quintile (least deprived)	45.8%	168	*Ref*.	P = 0.024	156	*Ref*.	p = 0.530
4^th^ Quintile	40.0%	155	0.79 (0.51–1.23)		144	0.77 (0.46–1.30)	
3^rd^ Quintile	41.1%	166	0.82 (0.53–1.27)		140	0.79 (0.46–1.36)	
2^nd^ Quintile	32.6%	227	0.57 (0.38–0.86)		201	0.66 (0.39–1.12)	
1^st^ Quintile (most deprived)	31.9%	204	0.55 (0.36–0.84)		171	0.60 (0.32–1.12)	
Health region where patient lived							
Health region 1	43.5%	260	*Ref*.	p = 0.005	247	*Ref*.	p = 0.988
Health region 2	32.3%	198	0.62 (0.42–0.91)		169	0.92 (0.52–1.63)	
Health region 3	39.1%	297	0.83 (0.59–1.17)		268	0.97 (0.65–1.44)	
Health region 4	32.1%	165	0.62 (0.41–0.93)		128	1.00 (0.58–1.74)	
Type of cancer (underlying cause of death)							
Digestive organs	37.9%	256	*Ref*.	p = 0.414	222	*Ref*.	p = 0.513
Respiratory and intra-thoracic organs	37.2%	199	0.97 (0.66–1.42)		176	1.03 (0.65–1.62)	
Eye, brain and other parts of CNS	36.8%	19	0.96 (0.36–2.51)		18	1.50 (0.50–4.52)	
Breast	35.9%	78	0.92 (0.54–1.56)		74	1.16 (0.62–2.17)	
Lymphoid/haematopoietic/related tissue	30.1%	73	0.71 (0.40–1.24)		64	0.58 (0.29–1.15)	
Male genital organs	40.0%	75	1.09 (0.65–1.85)		67	0.97 (0.50–1.86)	
Female genital organs	28.2%	39	0.64 (0.31–1.35)		34	1.07 (0.46–2.48)	
Urinary tract	42.2%	45	1.20 (0.63–2.28)		39	1.35 (0.63–2.90)	
Lip, oral cavity and pharynx	17.6%	17	0.35 (0.10–1.25)		15	0.50 (0.10–2.51)	
Melanoma and skin	42.9%	14	1.23 (0.41–3.65)		13	1.13 (0.31–4.17)	
Uncertain/unspecified/other	46.7%	105	1.43 (0.91–2.27)		90	1.63 (0.94–2.84)	
Place of death							
Home	32.6%	193	*Ref*.	p = 0.369	183	*Ref*.	p = 0.162
Hospital	39.9%	336	1.37 (0.94–1.99)		297	1.54 (1.00–2.38)	
Hospice	37.4%	313	1.23 (0.84–1.80)		271	1.28 (0.82–1.99)	
Nursing home	41.0%	78	1.44 (0.84–2.47)		61	0.93 (0.47–1.84)	
Gender of proxy							
Male	30.1%	429	*Ref*.	p<0.001	429	*Ref*.	p = 0.007
Female	44.1%	383	1.84 (1.38–2.45)		383	1.58 (1.14–2.20)	
Proxy’s relationship to patient							
Spouse or partner	54.0%	202	*Ref*.	p<0.001	202	*Ref*.	p<0.001
Son or daughter	26.8%	429	0.31 (0.22–0.44)		429	0.26 (0.18–0.40)	
Brother or sister	43.7%	71	0.66 (0.38–1.14)		71	0.88 (0.48–1.61)	
Parent	33.3%	9	0.43 (0.10–1.75)		9	0.89 (0.17–4.84)	
Niece/nephew	51.1%	47	0.89 (0.47–1.68)		47	0.59 (0.28–1.22)	
Grandchild	28.6%	14	0.34 (0.10–1.12)		14	0.19 (0.05–0.70)	
Other	31.9%	47	0.40 (0.20–0.78)		40	0.25 (0.11–0.56)	
Days from death to contact							
110–150	37.1%	143	*Ref*.	p = 0.950	128	*Ref*.	p = 0.474
151–180	37.4%	163	1.02 (0.64–1.62)		145	1.08 (0.62–1.87)	
181–210	34.6%	136	0.90 (0.55–1.46)		124	0.79 (0.44–1.42)	
211–240	38.6%	153	1.07 (0.67–1.71)		132	1.10 (0.63–1.93)	
241–270	41.7%	132	1.21 (0.75–1.97)		125	1.43 (0.81–2.53)	
271–300	37.5%	144	1.02 (0.63–1.64)		115	0.90 (0.50–1.63)	
301–330	35.4%	48	0.93 (0.47–1.84)		43	0.75 (0.33–1.69)	

*Abbreviations*: UK, United Kingdom; IMD, Index of Multiple Deprivation; CNS, Central Nervous System; OR, odds ratio; AOR, adjusted odds ratio; CI, confidence intervals

*All characteristics of the patients and the informants of death, including timing of contact were entered simultaneously in the regression model (N = 812, excluding 108 cases with missing data, i.e. 11.7% of all 920 cases). Model-fitting statistics: Hosmer and Lemeshow (*Χ*^*2*^ *= 7*.*883*, *p = 0*.*445*), Nagelkerke R2 (20.3%). 70.1% of the overall cases were correctly predicted (41.0% of active refusals and 87.1% of silent refusals). AUC: 0.732.

Multivariate analysis using logistic regression showed that the patient’s age, proxies’ gender, relationship to patient and patient’s country of birth were independently associated with actively refusing to take part in the survey (Table 4). Informants of patients aged 70+ (AOR ranged from 3.42 for the 70–79 age group to 6.59 for the 90+ age group) and female proxies (AOR 1.58; 95%CI:1.14–2.20) were significantly more likely to give an active refusal. The odds were lower if the informant was a son/daughter (AOR 0.26; 95% CI:0.18–0.40), a grandchild (AOR 0.19; 95% CI:0.05–0.70) or other (AOR 0.25; 95% CI:0.11–0.56) or if patient was born overseas (AOR 0.49; 95%CI:0.32–0.77). Area social deprivation and health region lost statistical significance after adjusting for other factors. Timing since death, patient’s gender, place of death, and type of cancer were not associated with the provision of active refusals.

### 5 Reasons for refusal

From the 348 nonparticipants who actively refused to take part, 205 (58.9%) justified their decision. Of these, 80.0% wrote their reasons in the reply slip (n = 164), 17.6% (n = 36) gave reasons over the phone and 2.4% (n = 5) wrote a letter to the research team. In total, 350 refusal reasons were given, with 38.1% (n = 78) giving more than one reason (range 1 to 7 reasons).

Sixty-four percent of the nonparticipants who provided reasons for refusal were women; 75.6% were proxies of patients aged 70–90+ years old, 40.5% were informants for patients who had died in a hospital and 33.7% for patients who had died in a nursing home.

Through content analysis, we identified seven categories of reasons for refusal (study-related, proxy-related, grief-related, life-related, care-related, non-specific and other) and 34 subcategories (Table 5). Coding agreement between raters was almost perfect (≥.81) for 23 sub-categories, substantial (≥0.61 & ≤0.80) for eight sub-categories, moderate (≥0.41 & ≤0.60) for two and poor (<0.00) for one sub-category (‘feels nothing more to be said about the care received’).

**Table 5:** Classification of reasons for refusal.

Categories	Sub-categories	n*	kappa	*n* disagreements
Study-related	**Total study-related**	**126**		
	Questions not applicable (e.g. died quickly, complex case, treated privately)	22	0.901	3
	Questions upsetting/stressful/intrusive/insensitive	18	0.852	5
	Approach or correspondence upsetting/”crossed”/ no further contact/off the list	18	0.906	3
	Prepared or tried to fill in but was not able to	16	0.797	6
	Questions bring it all back, bring memories	15	0.836	4
	Length of questionnaire/ too many details/too many boxes	13	0.847	4
	Feels study will not make any difference or has hidden agenda	12	0.954	1
	Questionnaire too complicated to complete (e.g. for elderly)	5	0.795	2
	Questions irrelevant, do not cover what person wishes to say	5	0.659	3
	Disapproves not being warned before receiving questionnaire	2	0.798	1
Proxy-related	**Total proxy-related**	**88**		
	Relative or friend with limited knowledge/involvement in care (e.g. not present)	21	0.894	4
	Professional, care home manager, lawyer, funeral officer (no family around)	19	0.942	2
	Limited knowledge/involvement but unclear if relative/friend or professional	14	0.856	4
	Potential respondent unwell, ill, disabled	9	0.870	2
	Cannot remember/recall requested information	9	0.870	2
	Does not wish to distress best person to answer	6	0.795	2
	Does not take part in surveys/does not want to fill in forms	5	0.828	2
	Death of potential participant	3	1.000	0
	Participation in another study	2	0.664	1
Grief-related	**Total grief-related**	**88**		
	Still grieving/still shocked/still raw/would be upsetting, distressing or painful	39	0.935	4
	Bad/traumatic/painful experience and times (not explicitly about care)	19	0.915	3
	Does not want to be reminded (e.g. go over, go back)	10	0.950	1
	Too soon, too early	9	0.895	2
	Too late, far down the line to go back over	6	0.907	1
	Too many things to sort out (e.g. deceased’s paperwork)	5	1.000	0
Life-related	**Total life-related**	**16**		
	Busy life, no time (e.g. kids, home to run)	5	0.907	1
	Busy caring for someone ill at moment	3	0.496	2
	“move on” events (e.g. moved house, had grandchild)	4	0.745	2
	“problematic” events (e.g. other relative died)	4	1.000	0
Care-related	**Total care-related**	**8**		
	Bad/traumatic care experiences	6	0.657	4
	Feels nothing more to be said about care received	2	-0.005	2
Non-specific	**Total non-specific**	**14**		
	Does not wish to/feel like doing it but no reason given	7	1.000	0
	Difficult/cannot manage/does not feel able but no reason given	7	0.870	2
Other	Any other reason not described above ^†^	10	0.502	17

* 205 non-participants volunteered reasons for refusal; N cells do not add to 205 as non-participants provided one reason (*n* = 127), two (*n* = 38), three (*n* = 22), four (*n* = 10), five (*n* = 6), six (*n* = 1) or seven (*n* = 1).

^†^ Included use of incorrect title in invitation letter (e.g. Ms instead of Mrs) (*n* = 2), NHS complaint procedure regarding care in process (*n* = 1), confidentiality and data safety concerns (*n* = 1), potential participant was Spanish and did not know how to complete the questionnaire (*n* = 1), passed questionnaire to patient’s partner (*n* = 1), was a social worker and “would say that palliative care was excellent”(*n* = 1), stated there was not enough contact with hospital (not clear if own or patient contact) (*n* = 1), patient residence outside studied areas (*n* = 1), asked other relative to help but person could not help either (*n* = 1).

The most common reasons were study-related (36.0% or 126 reasons). These referred to the informants feeling that the questions were not applicable to the patient’s case, the questionnaire was too long, questions were upsetting, or beliefs that the questionnaire would not make any difference. Proxy and grief related-reasons were the second most frequently mentioned reasons (each accounting for 25.1% of all reasons or 88 reasons each). Proxy-related reasons were provided by professionals who registered the death of a patient when there was no family around, relatives who felt they had no knowledge about the care received, those replying on behalf of their relatives who were the main carers, or the main carers themselves stating they were disabled/fragile and could not therefore complete the questionnaire. The most common grief-related reasons were from nonparticipants stating that they were still grieving and it would be too upsetting or painful to take part. Other reasons for refusal were life-related (4.6% of all reasons), such as being too busy to take part; care-related (2.3%) such as stating that patient received good care, and non-specific (4.9%). The latter referred to nonparticipants simply stating that they did not wish to take part. Less than 3.0% of reasons were coded as “other” and these are fully described in Table 5. Examples of nonparticipant quotes by main categories are shown in Table 6.

**Table 6:** Examples of reasons for refusal by main category*.

**STUDY-RELATED REASONS**	“The questions asked are intrusive and upsetting. Asking questions like *‘how did you feel in the last week of his life’*? How do you think we felt?”
	“I felt the questionnaire was too long and some questions were too similar. Also frustrating was a lot of questions weren't relevant to my family member's death. The three month time span placed also made the survey awkward as she was only aware of her condition for a few weeks”
	“If I thought the questionnaire would make any difference, I would fill it out”
	“It is very difficult to fill in this form as my mother-in-law had for the last 3 years of her life been blind, practically paralysed in both arms and legs and unable to communicate, therefore I find that most questions contradict themselves in one way or another”
	“My mother was in a coma for the last 6 months of her life which was spent in a nursing home. As most of the questions did not apply to her I did not think it would be much help to you”
	“The questionnaire does not cover the things that I would wish to say related to the death of my late wife [name]. I have only admiration for the treatment she received at hospital [name]. Her treatment at hospital [name] was the complete reverse. I would however be happy to converse with someone from your organisation on a one to one basis be it via a telephone conversation or a face to face interview”
**PROXY-RELATED REASONS**	“I am not the best person to complete this questionnaire, as I only visited my uncle once in hospital before his death. The people who helped and visited him a lot are friends (locally). I do not have their addresses”
	“We are only the solicitors dealing with the Estate of the deceased. We did not have any involvement with his care”
	“I am sorry to say this survey is beyond my mother who is now 84 and struggling with breast cancer following my father's death. These surveys are far too complicated for some elderly people”
	“I passed the original questionnaire to my sister as she was [name]'s partner and it seemed most appropriate for her to take part. I don't really want to keep asking her about whether she wants to take part as I imagine it would be a difficult thing for her to do so not sure if you'll get a response”
**GRIEF-RELATED REASONS**	“It has not been a year yet since I lost my loved one and am too upset at present to talk about them”
	“I just feel it is too early as I am still grieving”
	“I am having great difficulty coping with the loss of my husband please do not send any more mail to me”
	“It is still painful to sit and fill in questions of such a private time, I am not emotionally ready”
**LIFE-RELATED REASONS**	“I work full-time and have children and a home to run and as much as I would like to help I just don't have the time. Apologies”
	“I am very sorry I am not able to answer your questionnaire. I have been and still am caring for my husband 24/7. He is aged 91 years and he needs me”
**CARE-RELATED REASONS**	“Having lost my dear brother at the age of 55 years in hospital, who died like a dog and was treated like one for most of his life (…) Therefore I do not feel I can take part”
	“She was wonderfully looked after, [I have] nothing to comment more on the care provided to her”
**NOT SPECIFIC**	“I don't wish to, thank you, I don't want to be involved in this”
**OTHER REASONS**	“I do not trust any government department whatsoever to keep information confidential, safe or secure. Therefore I will not take part in your survey”

*To show quotes by main categories we did not give examples of multidimensional reasons given by one single nonparticipant when these overlapped different main categories.

## Discussion

Using multivariate analysis of socio-demographic population-based data, the QUALYCARE study identified important factors associated with participation in a postal mortality followback survey. We found that deaths in hospital and younger adult patients were underrepresented. We also found that similar factors were associated with both participation and giving an active refusal, except for country of birth which was only significant for the latter. Women and proxies of older patients were more likely to participate in the survey and also more likely to actively refuse when they decided not to take part. Likewise, relatives other than the spouse/partner were less likely to participate and also less likely to actively refuse when the decided not to participate. Through content analysis, we identified reasons for refusal and found that these were often multidimensional. Although grieving was an important reason, other issues related to the study itself and its design were also prominent. This suggests scope for improvement and potential increase in RRs.

The study’s RR is low compared to bereavement studies carried out in Italy (65%RR) [22], the United States (65% cooperation rate) [1] and the two recent national bereavement surveys carried out in England (46% RR for each) [2,55], but it is in line with many others [23–26,35,37,56,57] and 1% higher than we had estimated. Although we followed evidence-based strategies to increase RRs (e.g. follow-up contact, provision of second copy of questionnaire and use of a personalised questionnaire) [17], the topic was sensitive and we used a long questionnaire to meet our research aims. In addition, QUALYCARE was conducted in London, the UK city with the highest proportion of ethnic minorities [58]. All these factors were previously found to affect RRs [1,12,20]. Nonetheless, in the case of this analysis, non-response posed no challenges; on the contrary, it made the regression analyses easier because there was a reasonable number of “events” (i.e. non-participation) to do the modelling.

QUALYCARE was especially funded and designed to investigate the care provided to patients with cancer. This is a common cause of death (29% in 2013) in England and Wales [59] and was responsible for 25% of all deaths in Europe in 2010 [60], but as a consequence patients who died from conditions other than cancer were not included in the study. People dying from non-malignant conditions have a less predictable illness trajectory and are less likely to access palliative care services, for example [61–63]. It is therefore possible that people’s experiences of care and participation in research differ from those affected by cancer. Any transferability of our results to conditions other than cancer needs to be carefully considered. Subsequent to the present study, Evans et al. have adapted the QUALYCARE methodology to survey the end of life care provided to people dying from non-malignant conditions [64]; their analysis of non-response will help shed light on the factors associated with participation, active refusals and reasons for not taking part in a similar mortality followback survey within this patient group.

### 1 Factors associated with participation and providing active refusal

Since London is an urban area with an ethnically diverse population caution is needed when generalising results to other areas. Another limitation is that QUALYCARE is a cross-sectional survey, and any found associations do not imply causality. Finally, our regression models were not able to explain much of the variance neither in participation nor in the provision of active refusals. Other factors that we did not include in our models (such as complicated grief or depression) could possibly have played a role.

#### 1.1 Patient age

We found that proxies of older patients were more likely to take part in the study and to give an active refusal. This supports previous findings that older age (from patients) is associated with increased odds of participation [21,24,27–29]. Reasons for this are not fully understood. Perhaps death is less of a shock when patients are older and as a consequence people feel more able to take part (or to contact the research team to let them know they would not like to participate if that is the case). Our results suggest that QUALYCARE data might be more relevant to the care provided to older people. This could limit the sample representativeness, but can provide crucial evidence for end-of-life care, as populations in need get older [65].

#### 1.2 Relationship to the deceased and proxy gender

Relatives other than the spouse/partner were less likely to take part in our study, in contrast with a similar survey in Italy (ISDOC study) which found that sons and daughters were more likely to take part [22]. Family structures in Italy might help to explain these differences (e.g. strong family support and extended families living together) [66]. In the UK, carers looking after someone in the same household are more likely to be spouses or partners [67]. However, sons and daughters were the largest group among the informants of death in our study, and less likely to respond than spouses and partners. This result may be because sons and daughters may have a number of family responsibilities that precludes them from taking part in research as much as spouses and partners. They might also live in a different household (or be less involved in the care provided to the patient) compared to the patient’s spouse or partner. It is possible that the response rate would have improved if more spouses or partners were involved. However, cancer is a condition strongly associated with older age [68], so it is reasonable to expect that a large number of informants will be sons and daughters.

We also found that female proxies were more likely to participate, opposite to findings from other studies [22,27,29]. When women did not take part, they were more likely to provide active refusals. The association was present even after accounting for the relationship to the deceased. Research evidence on grief suggests that men and women grieve differently, and that men are less likely to talk about their feelings and experiences [69]; this might help explain our results.

#### 1.3 Place of death

Proxies of patients who died in hospital were less likely to return a completed questionnaire compared to proxies of patients who died at home. Our results are similar to those from the ISDOC study in Italy [22]; other studies in the UK also found higher response for proxies of patients who died at home [24,43,57]. None of these studies, however, discussed explanations and it is possible that the reasons are multifactorial. Importantly, in the main analysis of the QUALYCARE study we found that a home death was more peaceful for patients than death in hospital and resulted in less intense grief for proxies, after adjusting for confounders [70]. This is consistent with evidence stating that people experiencing fewer problems and having more positive views about the care received may be more likely to return a completed questionnaire [71]. It is also possible that proxies of patients who died at home knew more about the care provided and felt more able to contribute to the study.

#### 1.4 Social deprivation and country of birth

Social deprivation was associated with participation in univariate analysis, but lost statistical significance after adjusting for confounding factors. This might have occurred because people living in deprived areas are more likely to die at a younger age and in hospitals [72]. Both patient’s age and hospital death were associated with participation in our study. In our multivariate analysis investigating factors associated with active refusal, deprivation also lost statistical significance and this may be because patients born overseas (whose proxies were less likely to give active refusals) usually live in more deprived areas, especially in inner London [58]. Our findings suggest that other factors might play a bigger role than deprivation itself, but any conclusions need to be seen with caution and the topic warrants more research. Furthermore, deprivation is an area level variable rather than an individual level variable and there is the risk of ecological fallacy. Those living in a more deprived area are not necessarily deprived. Likewise, those who live in a less deprived area can actually be deprived. This is especially true in London, where health regions can have an unusual spatial distribution of both very affluent and very poor residents [73].

Proxies of patients who were born overseas (as opposed to being born in the UK/Ireland) were more likely to provide an active refusal. Almost half of the proxies of patients who were born overseas (139/287) were nonparticipants who did not actively refuse to take part. That means not only we have less knowledge about the care this patient group received, but we also do not know why their proxies did not participate. This could be due to several reasons (i.e. language barriers, different ways of grieving). Adding a note to the back of the questionnaire in different languages so potential participants know that they can have a translated version if required may be a helpful approach. Reaching out for these groups is a necessity in future research to help provide the best possible care for all, regardless of their country of origin or background.

#### 1.5 Timing of contact

Interestingly, timing of contact was not a significant factor in our study, contrary to findings from the ISDOC study in Italy [22]. This was the only study with bereaved relatives we could identify which also investigated the association of timing of contact with participation using multivariate analysis. Authors found that an increased interval from death to receiving the questionnaire made participation less likely. In QUALYCARE, although the majority of participants returned questionnaires 4–10 months after the patient’s death, 97 questionnaires were returned 11 to 12 months after the patient had died (six questionnaires were returned even later than that). Furthermore, eight potential participants who had refused to take part changed their minds and later returned a completed questionnaire. Perhaps this is a benefit of postal surveys; they give people more time to consider taking part than they would have on a face-to-face contact, and also more time to think about what the questionnaire requires and whether to respond to it.

### 2 Reasons for refusal

Our content analysis of reasons for refusal was limited by the comments we received. Since most of them were brief we were not able to carry out an in-depth analysis. Furthermore, although our study shed light on reasons why people did not take part, reasons for nonparticipation were unknown for the majority of nonparticipants (77.7% or 715 non-participants). This group includes 143 proxies who actively refused to take part without giving any reasons and 572 silent refusals. Their reasons might differ from the ones we received. It is also worth noting that the reasons for refusal provided over the telephone were not transcribed verbatim as conversations were not recorded. It is possible that researcher bias happened as a consequence.

Our findings show that more than a third (37.8%) of nonparticipants provided an active refusal, and more than half (58.9%) of those justified their reasons. Including reply slips in questionnaire packs seems to be an effective strategy to understand better the reasons for not taking part in cancer mortality followback surveys.

As expected based on the literature, grief is an important reason for refusal [22,36–39]; although in our study it was not the most common reason (accounting for a quarter of all reasons provided). This suggests that no matter how sensitively developed a questionnaire is, some potential participants will just not feel ready to respond. It is also possible that grief was underestimated as a refusal reason as people experiencing intense grief might be among those who did not contact the research team in any way.

Refusal reasons related to a perceived lack of knowledge about the care provided points to a limitation of using death registration data, since not all who register someone’s death have information about the care received by the patient. On the other hand, this suggests that the completed questionnaires can provide a more accurate picture of people’s experiences (since participants would have felt that they had sufficient knowledge to complete them). However, this also indicates that the study might be underrepresenting patients without informal carers or family around.

Proxy-related reasons also raise the issue about contacting informal carers who are sometimes fragile and disabled. In the context of an ageing population [65], this is likely to become a more common pattern in the future. The need to develop simpler, shorter questionnaires that are not burdensome to informal carers is then even more urgent. Questionnaire length was also highlighted as a study-related reason for refusal. Nonetheless, it is possible that a short questionnaire could be perceived by some as not doing enough justice to a life event as meaningful and salient as the end of life care received by a close one. Emphasising the importance of the study (and how it might benefit other patients and families in the future), working carefully on sensitive questions and leaving scope in the questionnaire for different care situations should also be a way forward. This can be challenging depending on the study aims (and when trying to develop shorter surveys).

### 3 Implications for research

Although the QUALYCARE study shed light on factors associated with participation while accounting for a number of potential confounders, further multivariate analyses of similar studies are still needed.

The need for more research is especially critical regarding the views and experiences of patients dying in hospitals and those without informal carers. Better representativeness might be achieved by using different proxies (such as care staff instead of close friends or relatives), by having questionnaires translated to different languages, being culturally sensitive when developing tools, among others. Targeted prospective studies instead of mortality followback surveys might also be more appropriate to reach out for these groups. Results from mortality followback surveys focused on non-cancer will be crucial to build evidence on non-response for proxies of patients with non-malignant conditions. Finally, investigating effective ways to reduce the questionnaire length in future studies may help to improve response rates.

## Conclusion

In summary, our results show that proxies of older patients, female informants and patient’s spouses were more likely to take part in a postal mortality followback survey designed to assess quality of end of life care provided to patients dying from cancer, whilst patients dying in hospitals were underrepresented. We also had little information about reasons for non-participation from proxies of patients who were born overseas. Changes to the study design and methods might help to increase RRs (as study-related reasons were the most commonly mentioned reasons for refusal), but it is important to be aware that reasons for refusal are often multidimensional and that grief is a common reason that must be accounted for and respected.

We used a robust, validated methodology to survey bereaved relatives and had a powered sample of a population dying from cancer in a metropolitan area in the UK. Our findings add much needed evidence to the field of end-of-life care studies with bereaved relatives. However, we still need further powered studies that use robust research methods. We also need to investigate different approaches which might be more appropriate to reach underrepresented groups.

## Supporting Information

S1 Fig QUALYCARE study reply form. (TIF)

S2 Fig QUALYCARE study call recording form. (TIF)

S1 Table Completed Strobe checklist for cross-sectional studies. (DOC)

## Abbreviations

AOR: adjusted odds ratio
CI: Confidence Intervals
IMD: Index of Multiple Deprivation
ONS: Office for National Statistics
UK: United Kingdom
