# Supplementary figures and images for: Factors Associated with Participation, Active Refusals and Reasons for Not Taking Part in a Mortality Followback Survey Evaluating End-of-Life Care
Source: PLoS One. 2016 Jan 8;11(1):e0146134. doi: 10.1371/journal.pone.0146134 (PMC4706352; doi:10.1371/journal.pone.0146134)

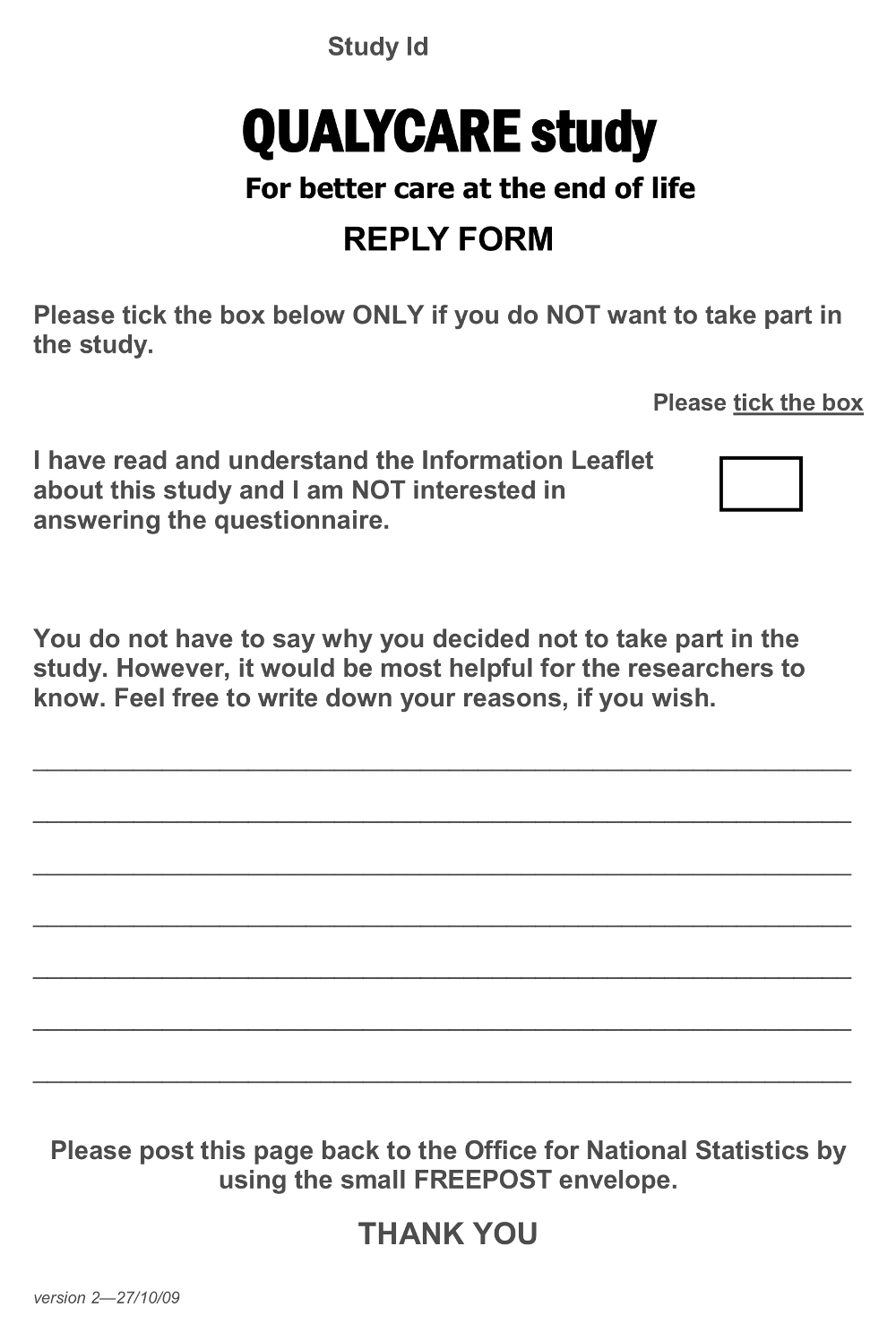

Supplement: S1 Fig — (TIF) [file pone.0146134.s001.tif]

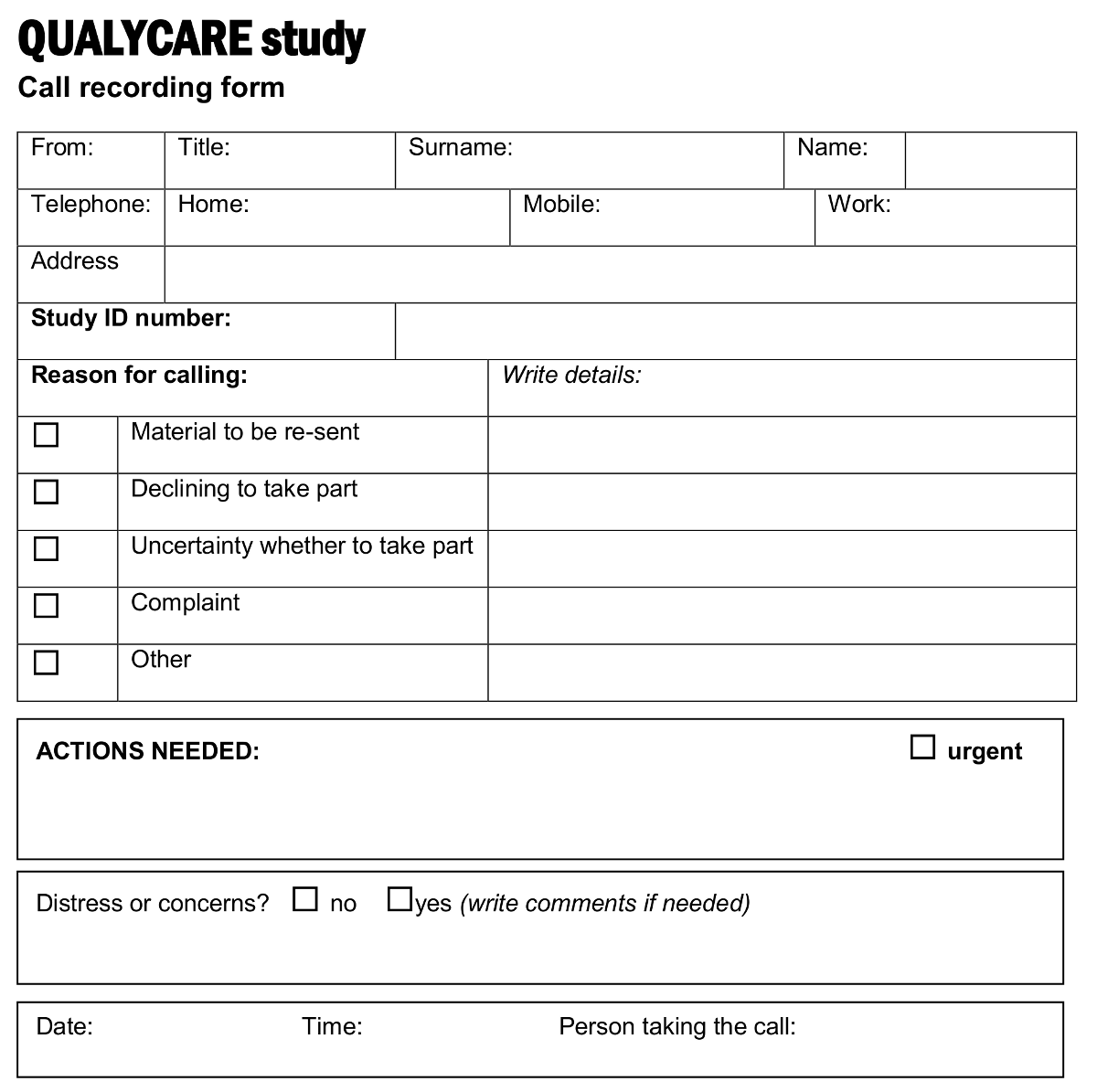

Supplement: S2 Fig — (TIF) [file pone.0146134.s002.tif]
