# Supplementary material for: Factors Associated with Participation, Active Refusals and Reasons for Not Taking Part in a Mortality Followback Survey Evaluating End-of-Life Care
Source: PLoS One. 2016 Jan 8;11(1):e0146134. doi: 10.1371/journal.pone.0146134 (PMC4706352; doi:10.1371/journal.pone.0146134)
Supplement: S1 Table — (DOC) [file pone.0146134.s003.doc]

S1 Table – Completed Strobe checklist for cross-sectional studies

|  | Item No | Recommendation | Authors’ comments |
| --- | --- | --- | --- |
| **Title and abstract** | 1 | (*a*) Indicate the study’s design with a commonly used term in the title or the abstract | This was done in the both sections; the study is a mortality followback survey (title and abstract) which was sent by post (in abstract, but not in title due to limited word count) |
|  |  | (*b*) Provide in the abstract an informative and balanced summary of what was done and what was found | The abstract succinctly describes the study aims, methods, results, study limitations and conclusions. |
| Introduction | | |  |
| Background/rationale | 2 | Explain the scientific background and rationale for the investigation being reported | This was done in the introduction. Although mortality followback surveys are widely used, multivariate analysis of nonresponse and systematic analyses of reasons for nonparticipation are rare. This study aimed to bridge this gap. |
| Objectives | 3 | State specific objectives, including any prespecified hypotheses | Specific objectives are shown both in the abstract (Background) and the last paragraph of the Introduction section |
| Methods | | |  |
| Study design | 4 | Present key elements of study design early in the paper | This was done in the “Materials and Methods” section, in the subsection “Study design and setting”. The study was a mortality followback survey, and a reference to the study protocol is provided |
| Setting | 5 | Describe the setting, locations, and relevant dates, including periods of recruitment, exposure, follow-up, and data collection | This was added to the “Materials and Methods” section, in the subsection “Participants and sampling” |
| Participants | 6 | (*a*) Give the eligibility criteria, and the sources and methods of selection of participants | This was added to the “Materials and Methods” section, in the subsection “Participants and sampling”. Figure 1 also provides additional information on eligibility criteria and the methods adopted for selecting participants. |
| Variables | 7 | Clearly define all outcomes, exposures, predictors, potential confounders, and effect modifiers. Give diagnostic criteria, if applicable | Not applicable. Reference to the study protocol is provided; this manuscript focuses on analysing factors associated with participation and the provision of active refusals, in addition to analysing refusal reasons. |
| Data sources/ measurement | 8* | For each variable of interest, give sources of data and details of methods of assessment (measurement). Describe comparability of assessment methods if there is more than one group | Data sources are described in the “Data Collection” section. These refer to the study questionnaire (and its components), reply slips and socio-demographic information provided by the Office for National Statistics |
| Bias | 9 | Describe any efforts to address potential sources of bias | This is described in the section “Statistical Analysis”. Multivariate analysis was used to analyse factors associated with participation and the provision of active refusals. Systematic analysis of reasons for non-participation was done independently by two researchers. These measures were chosen in order to deal with bias and confounding. |
| Study size | 10 | Explain how the study size was arrived at | This is summarised in the “Materials and Methods” section, in the subsection “Participants and sampling”. A reference to the study protocol (which has more detailed explanation) is also provided. |
| Quantitative variables | 11 | Explain how quantitative variables were handled in the analyses. If applicable, describe which groupings were chosen and why | This is described in the section “Statistical Analysis”, where tests carried out for which type of variable are specified. |
| Statistical methods | 12 | (*a*) Describe all statistical methods, including those used to control for confounding | This is described in the section “Statistical Analysis”, where the type of multivariate analysis (to control for confounding) carried out is specified. |
|  |  | (*b*) Describe any methods used to examine subgroups and interactions | The section “Statistical Analysis” described which groups were analysed and which methods were adopted; sensitivity analysis and study of interactions were not applicable. |
|  |  | (*c*) Explain how missing data were addressed | The section “Statistical Analysis” described the approach used to deal with missing data (all cases with missing data were excluded from multivariate analysis). Percentage of missing data by category and number of missing cases are shown as footnotes in all relevant tables. |
|  |  | (*d*) If applicable, describe analytical methods taking account of sampling strategy | Not applicable |
|  |  | (*e*) Describe any sensitivity analyses | Not applicable |
| Results | | |  |
| Participants | 13* | (a) Report numbers of individuals at each stage of study—eg numbers potentially eligible, examined for eligibility, confirmed eligible, included in the study, completing follow-up, and analysed | This is shown in Figure 1 (the study flowchart describing all stages of sampling and response) |
|  |  | (b) Give reasons for non-participation at each stage | Reasons for non-participation (when available) are described in “Results”, in the subsection “Reasons for refusal”. Examples of reasons provided are shown in Table 5. |
|  |  | (c) Consider use of a flow diagram | A flow diagram was adopted; this corresponds to Figure 1 |
| Descriptive data | 14* | (a) Give characteristics of study participants (eg demographic, clinical, social) and information on exposures and potential confounders | Characteristics of study participants and non-participants are shown in Table 1. Further characteristics of nonparticipants are shown in Table 3, while additional data on different types of participants (early, middle and late) are shown in Supplementary Table 1. Information on exposures was not applicable. All potential confounders were added simultaneously to the multivariate model, this is described in the “Statistical Analysis” section. |
|  |  | (b) Indicate number of participants with missing data for each variable of interest | Percentage of missing data by category and number of missing cases are shown as footnotes in all relevant tables. |
| Outcome data | 15* | Report numbers of outcome events or summary measures | Not applicable |
| Main results | 16 | (*a*) Give unadjusted estimates and, if applicable, confounder-adjusted estimates and their precision (eg, 95% confidence interval). Make clear which confounders were adjusted for and why they were included | Odds ratio, adjusted odd ratio and confidence intervals are provided in Tables 2 and 4. All socio-demographic variables were included in the model as they were potentially explanatory variables (this is explained in the “Statistical Analysis” section) |
|  |  | (*b*) Report category boundaries when continuous variables were categorized | These are described in the “Statistical Analysis” section |
|  |  | (*c*) If relevant, consider translating estimates of relative risk into absolute risk for a meaningful time period | Not applicable |
| Other analyses | 17 | Report other analyses done—eg analyses of subgroups and interactions, and sensitivity analyses | Not applicable |
| Discussion | | |  |
| Key results | 18 | Summarise key results with reference to study objectives | Key results were described in the first paragraph of the “Discussion” section |
| Limitations | 19 | Discuss limitations of the study, taking into account sources of potential bias or imprecision. Discuss both direction and magnitude of any potential bias | Limitations of the studies were described in the “Discussion” section (second paragraph); the subsection “Factors associated with participation and providing active refusal” (first paragraph); subsection “Reasons for refusal” (first paragraph); and also subsection “Implications for research” (first paragraph). |
| Interpretation | 20 | Give a cautious overall interpretation of results considering objectives, limitations, multiplicity of analyses, results from similar studies, and other relevant evidence | As above, results were interpreted in line with the study limitations. Results from similar studies are presented throughout the discussion section. A summary of results in line with our objectives is also shown throughout the “Discussion” section and also in the “Conclusion” section (first paragraph) |
| Generalisability | 21 | Discuss the generalisability (external validity) of the study results | Generalisability was discussed in the “Discussion” section, subsection “Factors associated with participation and providing active refusal” (first paragraph). |
| Other information | | |  |
| Funding | 22 | Give the source of funding and the role of the funders for the present study and, if applicable, for the original study on which the present article is based | Funding information was removed from the Acknowledgements section as per PLOS ONE’s guidelines. It was then provided online as part of the manuscript submission process. |

*Give information separately for exposed and unexposed groups.

**Note:** An Explanation and Elaboration article discusses each checklist item and gives methodological background and published examples of transparent reporting. The STROBE checklist is best used in conjunction with this article (freely available on the Web sites of PLoS Medicine at http://www.plosmedicine.org/, Annals of Internal Medicine at http://www.annals.org/, and Epidemiology at http://www.epidem.com/). Information on the STROBE Initiative is available at [www.strobe-statement.org](http://www.strobe-statement.org/).

**Manuscript:** *Factors associated with participation, active refusals and reasons for not taking part in a mortality followback survey evaluating end-of-life care*

Natalia Calanzani, Irene J Higginson, Jonathan Koffman, Barbara Gomes
